# Artificial intelligence and data analytics in human talent management

**DOI:** 10.3389/frai.2026.1793296

**Published:** 2026-05-15

**Authors:** Diana Alexandra Sarmiento Orna, Mariuxi Fernanda Apolo-Silva, Alvaro Paul Solís-Naranjo, Ely Israel Borja-Salinas, Eduardo Javier Espinoza-Solis, Dennis Alfredo Peralta-Gamboa

**Affiliations:** 1 Universidad Estatal de Milagro, Milagro, Ecuador; 2 Universidad Tecnica Estatal de Quevedo, Quevedo, Ecuador; 3 Universidad Estatal de Bolívar, Bolívar, Ecuador

**Keywords:** data-driven decision-making, digital transformation, emerging technologies, human resources innovation, intelligent automation, predictive analytics

## Abstract

**Introduction:**

Digital transformation has reshaped human talent management, with Artificial Intelligence (AI) and Data Analytics emerging as key tools for optimizing recruitment, retention, performance evaluation, and employee development. Despite growing interest, the literature remains fragmented across technical, managerial, and ethical perspectives. This study provides an integrated bibliometric and qualitative analysis of research on AI and Data Analytics in human talent management from 2015 to 2025, aiming to map evolution, thematic trends, methodological developments, and research gaps.

**Methods:**

A mixed-methods approach combined bibliometric analysis with qualitative synthesis. A structured search in Scopus and Web of Science retrieved 137 records using Boolean operators combining AI/machine learning terms with People/HR Analytics and talent management concepts. After removing duplicates and irrelevant studies based on predefined inclusion criteria (peer-reviewed articles and reviews in English or Spanish), a final dataset of 82 documents was analyzed. Bibliometric techniques in R (v4.4.2) and VOSviewer (v1.6.20) examined scientific productivity, citation patterns, collaboration networks, and keyword co-occurrence. Additionally, the 30 most cited articles underwent qualitative synthesis to extract key conceptual and methodological contributions.

**Results:**

Scientific production showed exponential growth, particularly after 2020, with peaks in 2024 (21 articles) and 2025 (27 articles). Research concentrated in India, Germany, the United States, and the United Kingdom, with limited contributions from Latin America and Africa. Four thematic clusters emerged: (1) People Analytics and process optimization, (2) predictive models and machine learning, (3) data governance and ethical considerations, and (4) convergence of AI, Big Data, and advanced analytics. Highly cited studies highlighted advances in predictive modeling for turnover and recruitment, while also emphasizing ethical risks such as algorithmic bias and the need for explainable AI (XAI).

**Discussion and conclusion:**

The field is evolving toward the integration of predictive capabilities with organizational decision-making and ethical frameworks, shifting human talent management from reactive to proactive, data-driven practices. However, gaps persist in empirical validation of models, standardization of ethical guidelines, interdisciplinary collaboration, and geographic diversity. This study contributes a unified socio-technical perspective that links technological innovation with organizational processes and governance. The findings offer a foundation for future research on context-specific, ethically grounded AI applications in emerging markets and support organizations in leveraging analytics for more adaptive and responsible talent management.

## 1 Introduction

Over the past decade, digital transformation has significantly altered the manner in which organizations manage human resources. The integration of Artificial Intelligence (AI) and Data Analytics has emerged as a fundamental component in optimizing processes related to recruitment, retention, performance evaluation, and employee development (Mir, 2024; Kadirov et al., 2024; Pessach et al., 2020; Rožman et al., 2022). This development addresses the imperative for companies to make data-driven decisions, enhance operational efficiency, and anticipate trends in the labor market.

The bibliometric analysis conducted in this study, encompassing the period from 2015 to 2025, indicates a significant exponential increase in scientific output, particularly from 2020 onwards. This trend underscores the heightened emphasis placed by academics and professionals on the integration of intelligent systems within human resource management. Furthermore, the study reveals that research efforts are predominantly concentrated in India, Germany, the United States, and the United Kingdom, whereas regions such as Latin America and Africa remain underrepresented. This disparity highlights a research gap that necessitates studies specifically tailored to emerging contexts.

The findings from the most cited articles reveal the consolidation of four main thematic areas:

1. People Analytics and internal process optimization (Cho et al., 2023; Margherita, 2022).
2. Predictive models and machine learning for anticipating workplace behaviors (Zhao et al., 2015; Sisodia et al., 2017).
3. Governance, ethics, and transparency in data use (Giermindl et al., 2022).
4. Integration of AI, Big Data, and advanced platforms (Berhil et al., 2020).

Recent research has expanded upon these perspectives. For example, Schinnenburg and Böhmer (2025) underscore the necessity of redesigning organizational strategies in the digital era to effectively address labor market volatility. Similarly, Tripathi et al. (2025) propose a machine learning-based model aimed at optimizing employee turnover prediction in highly dynamic organizations. Concurrently, Marabelli and Lirio (2024) investigate the role of artificial intelligence and the metaverse in fostering inclusive work environments, thereby introducing opportunities to enhance diversity, equity, and inclusion (DEI) within companies.

The development of models such as Large Language Models (LLMs) is significantly influencing the prediction of workplace behaviors. Ma et al. (2025) illustrate that architectures based on GPT-4 enhance the capacity to forecast employee attrition through the application of contrastive learning and ensemble learning techniques, thereby establishing new benchmarks in predictive analytics within personnel management.

These advancements have facilitated a transition for organizations from a reactive to a proactive paradigm, wherein data functions as the fundamental input for crafting individualized talent development strategies. Nevertheless, the swift integration of these technologies presents ethical and regulatory challenges. As Giermindl et al. (2022) observe, the growing dependence on opaque algorithms has the potential to exacerbate historical biases, thereby impacting fairness in selection and promotion processes. This has led to a renewed focus on explainable AI (XAI) models, which aim to harmonize technological innovation with transparency and social responsibility.

The accumulated evidence indicates that artificial intelligence and data analytics are fundamentally transforming the principles of human talent management, thereby promoting organizations that are more adaptive, efficient, and competitive (Pareek, 2024). However, there remain substantial deficiencies in the empirical validation of models, the regulation of ethical data usage, and the contextual adaptation of solutions in emerging markets. Addressing these challenges is essential for progressing towards an intelligent management model, wherein technology and the human dimension strategically complement one another.

Despite the growing number of studies on Artificial Intelligence and HR Analytics, the literature remains fragmented across technical, managerial, and ethical perspectives. Most existing studies focus either on technological development or organizational applications, without integrating these perspectives into a coherent conceptual framework.

Therefore, this study contributes to the literature in three main ways. First, it provides a systematic bibliometric mapping of global research on AI-driven talent management between 2015 and 2025. Second, it integrates bibliometric indicators with qualitative synthesis of the most influential studies, enabling a multidimensional interpretation of the field. Third, the study identifies emerging theoretical patterns linking predictive analytics, organizational decision-making, and ethical governance, thereby contributing to the conceptual development of AI-enabled human talent management.

## 2 Methodology

### 2.1 Study design

This study employs a descriptive, exploratory, and mixed-methods approach, integrating quantitative bibliometric analysis with a qualitative review of the most influential studies on Artificial Intelligence (AI), People Analytics, and human talent management.

The bibliometric analysis assesses scientific productivity, academic impact, international collaboration, and emerging thematic clusters (Indahsari et al., 2023; Vagelas and Leontopoulos, 2023).

The qualitative review concentrates on the 30 most cited articles to discern key findings, applied trends, and future challenges in the integration of AI and data analytics into talent management processes.

### 2.2 Database selection

The selected databases were Scopus and Web of Science (WoS) due to their extensive coverage, academic rigor, and reliability for bibliometric studies. The combination of both sources provides a broader view of the global research landscape on AI applied to human talent management.

### 2.3 Search strategy

The search equation was designed using Boolean operators and key terms to capture the maximum number of relevant publications.

The full search query applied in Scopus and Web of Science was as follows: in Scopus: TITLE-ABS-KEY((“Artificial Intelligence” OR “Machine Learning” OR “Deep Learning” OR “Generative AI”) AND (“People Analytics” OR “HR Analytics” OR “Human Resource Analytics” OR “Workforce Analytics”) AND (“Talent Management” OR “Human Capital” OR “Employee Retention” OR “Recruitment” OR “Upskilling”)) and WoS: TS = ((“Artificial Intelligence” OR “Machine Learning” OR “Deep Learning” OR “Generative AI”) AND (“People Analytics” OR “HR Analytics” OR “Human Resource Analytics” OR “Workforce Analytics”) AND (“Talent Management” OR “Human Capital” OR “Employee Retention” OR “Recruitment” OR “Upskilling”)).

### 2.4 Inclusion and exclusion criteria

The criteria were as follows: (1) Include peer-reviewed scientific articles, conference papers, and reviews; exclude conference proceedings, books, and book chapters; (2) Documents published in English and Spanish, and (3) Documents from all thematic areas and published in any year.

### 2.5 Data filtering process

**Figure 1:**
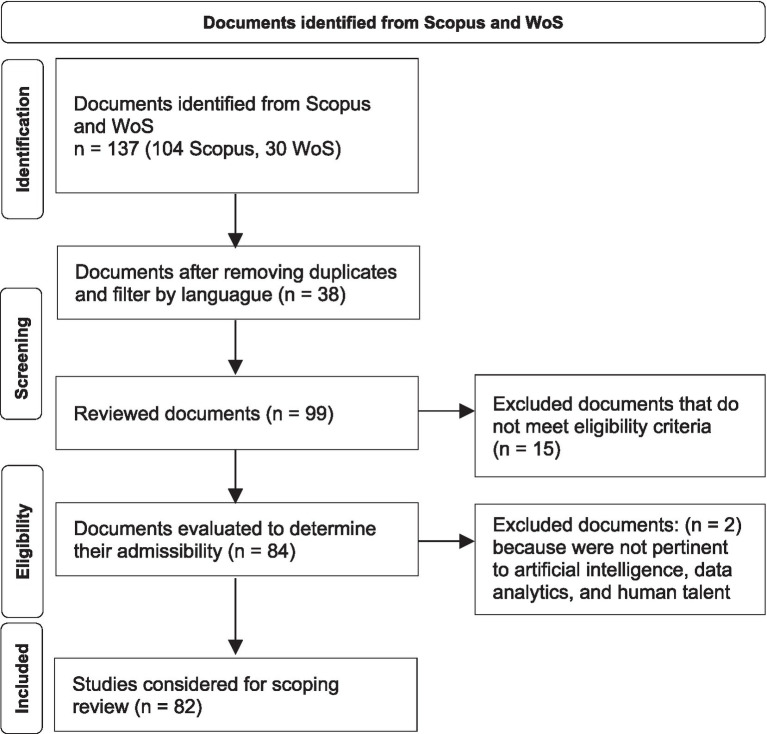
Data collection and refinement process.

As shown in Figure 1 the initial search, conducted on September 10, 2025, resulted in the identification of 33 records in the Web of Science (WoS) and 104 in Scopus. Following the application of inclusion and exclusion criteria, 33 records from WoS were downloaded in.xlsx format, and 85 records from Scopus in.csv format. The data were subsequently imported into R software (version 4.4.2), where titles were converted to lowercase using the tolower() function, facilitating the identification and removal of 19 duplicate records. Thereafter, titles, abstracts, and keywords were scrutinized using the grepl() function to exclude 17 publications that were not pertinent to artificial intelligence, data analytics, and human talent management. Ultimately, a total of 82 documents constituted the database employed for both quantitative and qualitative analyses.

After the screening and eligibility process, a final dataset of 82 documents was obtained for the bibliometric analysis. From this corpus, the 30 most cited articles were selected for qualitative synthesis because they represent the most influential contributions in the field. This dual approach allows the study to combine macro-level scientometric patterns with in-depth conceptual insights derived from highly cited research.

### 2.6 Bibliometric analysis

The quantitative analysis was conducted using the R programming (v4.4.2) and VOSviewer software (v1.6.20). Keyword co-occurrence analysis was performed using a minimum occurrence threshold of five keywords. Collaboration networks between countries and institutions were constructed based on co-authorship relationships identified in the bibliographic dataset. Thematic clusters were identified using VOSviewer’s clustering algorithm, which groups related keywords based on their co-occurrence strength within the literature. The main indicators include:

- Scientific Productivity: Number of articles published per year and production trends.
- Academic Impact: Total citations, average citations per article, and most cited articles.
- Journals and Quartiles: Identification of the most influential journals and their bibliometric metrics.
- Collaboration Analysis: Networks among countries.
- Thematic Analysis: Detection of keyword clusters and emerging trends through co-occurrence analysis.

### 2.7 Qualitative review

The 30 most influential articles were selected based on citation counts. For each study, the following elements were extracted:

- Main objective.
- Key findings and results.

This review complements the quantitative analysis, providing a comprehensive view of advances, limitations, and opportunities for improvement in AI-based talent management.

### 2.8 Analysis variables

The variables analyzed in this study, as shown in Table 1, include scientific production, impact, influential authors, leading journals, collaboration networks, and thematic trends, with specific indicators and tools for each dimension.

**Table 1:** Analyzed variables in the bibliometric study.

Dimension	Indicators	Tool
Scientific production	Documents per year	R
Impact	Total citations, average citations/article	R
Influential authors	Top 10 most productive authors	VOSviewer
Leading journals	Top 10 journals and quartiles	R
Collaboration	Networks among authors, institutions, and countries	VOSviewer
Thematic trends	Keyword clusters and emerging themes	VOSviewer

### 2.9 Integration with qualitative analysis

The integration of quantitative and qualitative results will enable:

- Mapping the main global trends in the application of AI to human talent management.
- Identifying research gaps and opportunities for innovation.
- Proposing a conceptual model on the interaction between AI, People Analytics, and strategic human talent management.

## 3 Results

### 3.1 Productivity and academic impact

**Figure 2:**
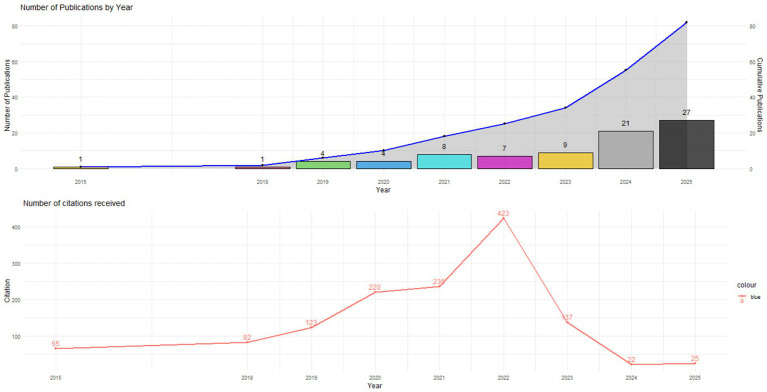
Annual evolution of scientific output and citations in artificial intelligence and data analytics in talent management (2015–2025).

Figure 2 depicts the temporal progression of scientific output and the citations accrued in the domain of Artificial Intelligence and Data Analytics as applied to human talent management over the period 2015–2025.

The findings indicate a consistent and exponential increase in the number of publications, contrasted by a more variable pattern in citation accumulation. During the initial years of analysis, from 2015 to 2018, scientific output was in its infancy, with merely one publication annually, indicative of the limited interest in the subject at that time. Beginning in 2019, an initial surge is observed with four articles, a trend that persists into 2020, coinciding with the integration of machine learning techniques and People Analytics into talent management processes.

In 2021, the number of publications rises to eight, followed by a slight decline in 2022 (seven articles). However, 2022 represents the peak in citations with 423, underscoring the high relevance and academic impact of the studies published during this period. This suggests that 2022 consolidated the most influential works in the application of predictive models and data analytics in human resources.

From 2023 onward, production continues to increase, reaching nine publications that year and showing significant growth in 2024 (21 articles) and 2025 (27 articles), establishing these two years as the most productive. However, in contrast to the rise in publications, citations experience a significant decline starting in 2023 (137 citations), reaching minimal values in 2024 (22 citations) and 2025 (25 citations).

This trend can be attributed to the effect of academic obsolescence, as recent articles have not yet had sufficient time to accumulate impact within the scientific community. Overall, the results indicate that research on Artificial Intelligence and Data Analytics in Human Talent Management is in a phase of accelerated expansion, with a significant increase in recent production but with impact concentrated primarily in studies published between 2020 and 2022. This finding confirms that the field is establishing itself as an emerging area of high relevance, with recent contributions laying the groundwork for future research directions and organizational applications.

### 3.2 Most influential journals

The selected documents were published in 40 distinct journals. Table 2 delineates the primary publication sources pertinent to research on Artificial Intelligence and Data Analytics as applied to human talent management, considering both the quantity of articles published and the citations accrued. The findings indicate a heterogeneous distribution in terms of productivity and scientific visibility, underscoring the prominence of certain high-impact journals in contrast to others with a greater number of publications.

**Table 2:** Leading scientific journals in artificial intelligence and data analytics research in talent management: productivity and citations received (2015–2025).

Source title	Documents	Citation
Lecture Notes in Networks and Systems	5	2
International Journal of Organizational Analysis	3	32
Human Resource Management Review	2	191
Decision Support Systems	1	200
European Journal of Information Systems	1	185
Indonesian Journal of Electrical Engineering and Computer Science	1	81
Proceedings of the National Conference on Artificial Intelligence	1	65
Journal of Management Analytics	1	62
Business and Information Systems Engineering	1	51

In terms of productivity, the journal Lecture Notes in Networks and Systems ranked highest, with five published articles. Nevertheless, despite its leading position in publication volume, the journal’s citation count is limited to two, indicating that these works may have a diminished academic impact and are likely to be focused on technical or preliminary contributions.

Conversely, several journals with a limited number of publications exhibit high citation impact. For example, Decision Support Systems (200 citations with a single article) and the European Journal of Information Systems (185 citations with one publication) illustrate that articles published in these journals possess substantial international visibility and significance within the scientific community.

The journal Human Resource Management Review is recognized as a highly influential source, with two articles garnering 191 citations, thereby establishing itself as a pivotal reference in the integration of People Analytics and strategic human talent management topics. Similarly, the International Journal of Organizational Analysis contributed three articles and 32 citations, representing an intermediate source that balances productivity with moderate impact.

Additional sources, including the Indonesian Journal of Electrical Engineering and Computer Science (81 citations), Proceedings of the National Conference on Artificial Intelligence (65 citations), Journal of Management Analytics (62 citations), and Business and Information Systems Engineering (51 citations), offer pertinent publications with considerable visibility, albeit less than that of the leading journals.

Overall, the results reflect a dual scenario.

- On the one hand, some journals have high productivity but low impact.
- However, there are sources with few publications but high academic recognition.

This pattern indicates that within the domain of research on artificial intelligence and data analytics in human talent management, the most significant advancements are predominantly found in highly cited articles published in specialized journals focused on management, organizational analytics, and decision support systems. This observation suggests that authors prioritize the dissemination of their findings in prestigious journals to influence and potentially transform practices in human capital management.

### 3.3 Analysis of collaboration between countries

**Figure 3:**
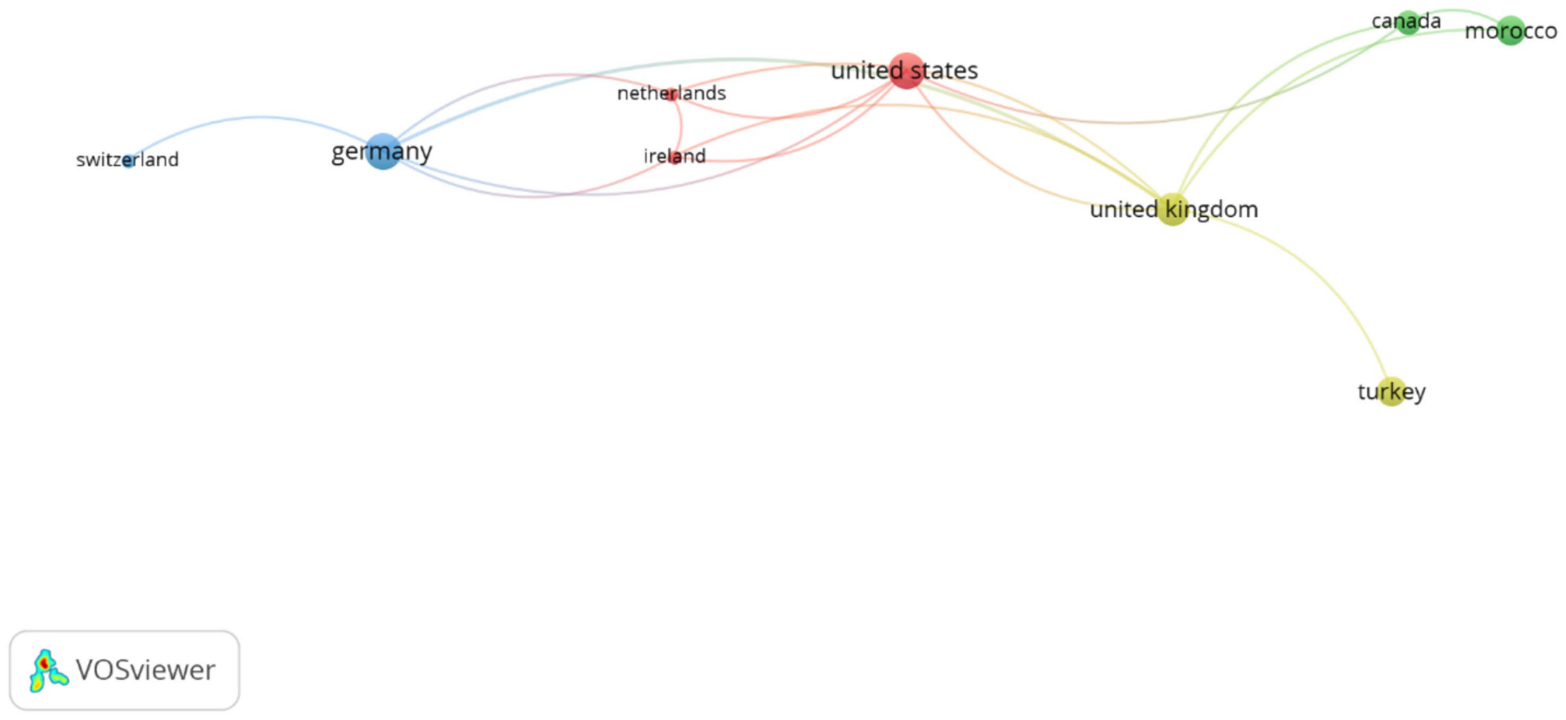
Map of international collaboration in research on artificial intelligence and data analytics in talent management (2015–2025).

Figure 3 depicts the international collaboration networks in research on Artificial Intelligence and Data Analytics applied to human talent management during the period 2015–2025. The results, supported by the data in the country production table, show a moderately concentrated collaboration structure, where certain countries act as central connectivity nodes and others exhibit limited or isolated participation.

### 3.4 Countries with the highest scientific productivity

India leads article production with 38 documents and 260 citations, although its international link strength (total link strength = 1) is low, indicating that much of its output is independent or locally collaborative. Germany (6 articles, 244 citations) and the United States (6 articles, 32 citations) followed, both with significant link strengths (6 and 5, respectively), reflecting their roles as strategic actors in global networks. The United Kingdom also holds a prominent position with five articles and a high link strength (8), serving as one of the main integrative nodes between Europe, the Americas, and Africa.

Meanwhile, Canada (three articles, 16 citations, link strength = 3) showed moderate participation, typically linked to collaborations with the United Kingdom and Morocco.

### 3.5 Countries with high citation impact

Some countries with low scientific production stand out due to the impact of highly influential individual articles:

- Israel (1 article, 200 citations).
- Italy (1 article, 191 citations).
- Switzerland (1 article, 185 citations).

These cases suggest highly relevant contributions to the methodological and conceptual development of the field, though without establishing active collaboration networks.

### 3.6 Strategic collaboration networks

The analysis of links between countries reveals three main cooperation clusters:

- European Cluster: Led by Germany, closely linked with Switzerland, the Netherlands, Ireland, and the United Kingdom.
- Anglo-Saxon Cluster: Headed by the United States and the United Kingdom, with significant connections to Canada and Morocco.
- Emerging Cluster: Comprising countries with moderate production, such as Turkey, the Philippines, Bangladesh, and Morocco, which are beginning to integrate into global networks.

### 3.7 Countries with marginal or isolated participation

Several countries with low scientific production and minimal or no link strength were identified, including China, Indonesia, Ukraine, and Uzbekistan, highlighting opportunities for future integration into international networks.

The results indicate that while scientific production on AI and data analytics in human talent management is increasingly global, international collaboration remains limited and concentrated among a few key actors. Countries such as Germany, the United Kingdom, and the United States lead in connectivity, whereas regions such as Asia and Latin America exhibit high levels of individual production but low internationalization.

This scenario reflects that the scientific community is in a consolidation phase, where strengthening interdisciplinary and intercontinental alliances is necessary to expand the scope and quality of research in this emerging field of study.

### 3.8 Thematic analysis

**Figure 4:**
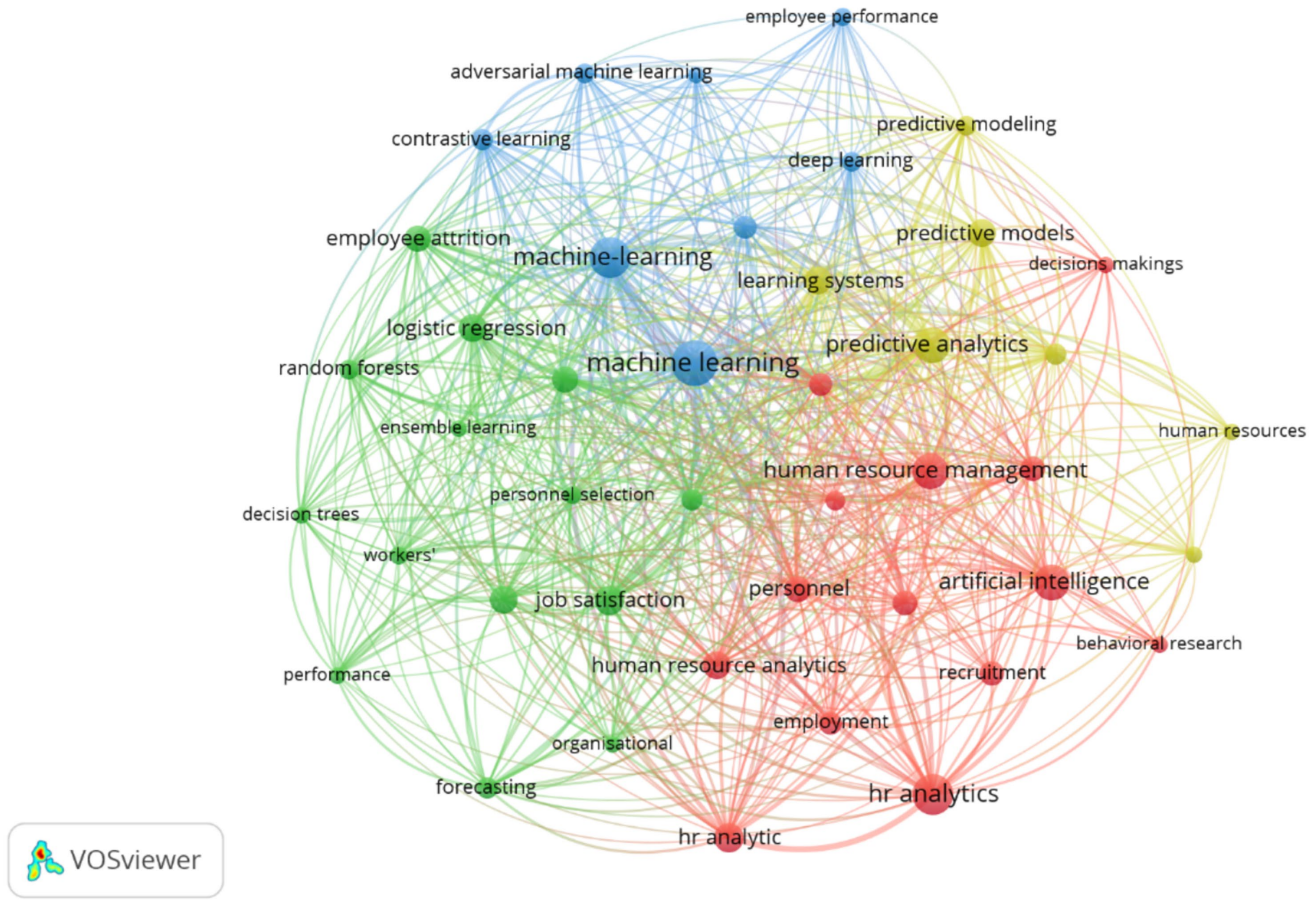
Map of keyword co-occurrence in research on artificial intelligence and data analytics in talent management (2015–2025).

Figure 4 presents the co-occurrence map of keywords generated using VOSviewer, identifying thematic relationships and conceptual clusters in research on Artificial Intelligence (AI) and Data Analytics applied to human talent management. The analysis, based on the frequency table and link strength, reveals four main clusters that structure the field of study.

### 3.9 Red cluster: strategic talent management and HR analytics (organizational focus)

This cluster integrates terms most directly related to human capital management and the use of People Analytics for decision-making. The most prominent keywords are:

- “HR analytics” (28 occurrences, link strength = 131)
- “Human resource management” (22 occurrences; link strength = 139) •
- “Predictive analytics” (22 occurrences; link strength = 155)
- “Talent management” (10 occurrences; link strength = 49)
- “Recruitment” (10 occurrences; link strength = 39)

This group reflects the growing adoption of predictive models, performance analytics, and talent retention techniques. It also indicates that research prioritizes data-driven strategies to optimize hiring, development, and employee retention processes.

### 3.10 Blue cluster: machine learning and predictive models (technical-scientific focus)

This cluster groups terms associated with the use of machine learning and deep learning to develop algorithms that enhance data analytics in human resources. The most relevant keywords are:

- “Machine learning” (35 occurrences; link strength = 245)
- “Machine-learning” (27 occurrences; link strength = 211)
- “Learning systems” (13 occurrences; link strength = 111)
- “Predictive models” (13 occurrences; link strength = 92)
- “Deep learning” (7 occurrences; link strength = 46)

This group highlights the strong integration between AI and talent management, where machine learning algorithms enable predictive models to assess performance, estimate turnover, improve recruitment processes, and support data-driven decision-making.

### 3.11 Green cluster: performance analytics and job satisfaction (behavioral and predictive focus)

This cluster links concepts related to individual performance, employee satisfaction, and performance optimization. The most prominent keywords are:

- “Job satisfaction” (15 occurrences; link strength = 109)
- “Employee retention” (13 occurrences; link strength = 72)
- “Employee turnover” (12 occurrences; link strength = 87)
- “Employee performance” (6 occurrences; link strength = 29)
- “Personnel selection” (6 occurrences; link strength = 43)

The findings indicate growing interest in understanding human factors that influence turnover, retention, and productivity, as well as in designing organizational policies to enhance the employee experience.

### 3.12 Yellow cluster: decision models and organizational behavior (complementary focus)

This cluster focuses on concepts linking decision-making with artificial intelligence and behavioral research. The most influential keywords are:

- “Decision making” (10 occurrences; link strength = 69)
- “Behavioral research” (5 occurrences; link strength = 22)
- “Resource allocation” (5 occurrences; link strength = 37)
- “People analytics” (9 occurrences; link strength = 40)

This group suggests an emerging emphasis on integrating behavioral metrics and advanced predictive models to support evidence-based human talent management.

The results demonstrate that research on AI and data analytics in human talent management is organized around four interconnected axes:

1. Organizational optimization through HR Analytics and People Analytics.
2. Development of advanced algorithms based on machine learning and deep learning.
3. Analysis of employee behavior and satisfaction to predict retention and turnover.
4. Enhancement of decision-making and resource allocation based on predictive models.

Collectively, Figure 4 reflects an interdisciplinary ecosystem where human resource management, artificial intelligence, and data science converge, positioning this field as a consolidating area of research with high applicability.

The thematic structure revealed by the keyword co-occurrence analysis suggests that research on AI-driven talent management is evolving along two complementary trajectories. On the one hand, there is a strong technological orientation toward predictive modeling and machine learning algorithms aimed at optimizing workforce management processes. On the other hand, an emerging line of research focuses on ethical governance, algorithmic transparency, and responsible data usage.

This dual trajectory reflects a broader transformation in human resource management research, where technological innovation increasingly intersects with organizational governance and social responsibility considerations.

### 3.13 Qualitative analysis

The qualitative synthesis focuses on the 30 most cited articles within the dataset because these studies represent the most influential contributions in the field and have shaped the theoretical and methodological development of AI-driven talent management research. Citation counts were used as the primary selection criterion to identify studies with the greatest academic impact. Although selecting the most cited articles may introduce a temporal bias favoring older publications, this approach was intentionally adopted to capture the most influential and theory-shaping contributions in the field. To mitigate this limitation, the bibliometric analysis includes the full dataset (82 documents), ensuring that recent trends and emerging topics are also represented. Therefore, the qualitative and quantitative components complement each other by balancing influence and recency.

The landscape of the 30 most cited studies, summarized in Table 3, shows a concentration of contributions in high-impact journals and consolidates research lines in prescriptive analytics, machine learning, and ethical data governance. Notable studies include those by Pessach et al. (2020), Margherita (2022), and Giermindl et al. (2022), distinguished by their citation volume and theoretical and applied influence.

**Table 3:** Synthesis of the 30 most cited studies on artificial intelligence and data analytics in human talent management (WoS, 2015–2025).

Authors	Journal/Source	Citations	Study objective	Main findings
Pessach et al. (2020)	Decision Support Systems	200	Propose a comprehensive analytics framework to support recruitment and staff allocation decisions.	Develops a predictive model and global optimization scheme that improves hiring efficiency.
Margherita (2022)	Human Resource Management Review	191	Conceptually define HR analytics and its main areas of application.	Identifies 106 key themes related to enablers, applications, and value creation in talent management.
Giermindl et al. (2022)	European Journal of Information Systems	185	Analyze the impact of People Analytics on human talent management.	Highlights benefits and risks of AI in HR, including biases, privacy, and ethical challenges.
Sisodia et al. (2017)		82	Explore the relevance of predicting employee turnover.	Emphasizes the financial impact of turnover and the need for efficient predictive models.
Berhil et al. (2020)	Indonesian Journal of Electrical Engineering and Computer Science	81	Analyze the application of HR Analytics and AI methods in human capital management.	Reviews techniques and predictive models that enhance strategic HR decision-making.
Zhao et al. (2015)	Proceedings of the National Conference on Artificial Intelligence	65	Improve skill characterization using NER and NEN.	Demonstrates how competency normalization enables more accurate predictive labor market analyses.
Gurusinghe et al. (2021)	Journal of Management Analytics	62	Explore the role of HR Analytics in strategic decision-making.	Shows how digitization and AI generate competitive advantages through data-driven decisions.
Köchling et al. (2021)	Business and Information Systems Engineering	51	Evaluate the impact of algorithmic decision-making in recruitment processes.	Concludes that algorithms reduce human biases but warns of potential indirect discrimination.
Chung et al. (2023)	Expert Systems with Applications	50	Analyze factors associated with employee turnover using AI models.	Early turnover prediction reduces costs and improves HR planning.
Arora et al. (2021)		49	Examine the integration of AI and HR Analytics to optimize HR management.	Highlights AI’s role in automation and improving organizational performance.
Cho et al. (2023)	Administrative Sciences	44	Review HR Analytics concepts and practices in the public sector.	Proposes a five-step process to implement analytics and improve personnel planning, selection, and evaluation.
Chakraborty et al. (2021)		38	Study employee turnover and its financial effects.	Concludes that turnover entails high costs and lost opportunities for organizations.
Islam et al. (2022)	South Asian Journal of Human Resources Management	30	Analyze factors influencing AI adoption in recruitment using the UTAUT model.	Survey of 283 professionals shows significant relationships between adoption and perceived credibility.
Shet and Nair (2022)	International Journal of Organizational Analysis	23	Propose the concept of ‘hiring quality’ using machine learning and P-E fit theory.	Uses a PAM clustering algorithm on IBM data to optimize candidate selection.
Sooraksa (2021)		17	Examine the evolution of HR Analytics over the past decade.	Highlights the importance of data-driven approaches for improving strategic HR decisions.
Escolar-Jimenez et al. (2019b)	International Journal of Emerging Trends in Engineering Research	16	Design a compensation algorithm using a neuro-fuzzy system for personnel selection.	Creates a salary matrix as a reference for performance management and salary reviews.
Marabelli and Lirio (2024)	Personnel Review	16	Explore opportunities and challenges of the metaverse in talent management.	Suggests the metaverse can enable fairer evaluations and enhance employee experiences.
Achchab and Temsamani (2021)		15	Analyze AI applications in HR and their challenges.	Presents strategies to strengthen the workforce using AI-based technologies.
Escolar-Jimenez et al. (2019a)	International Journal of Advanced Trends in Computer Science and Engineering	14	Improve candidate selection with a fuzzy logic-based pre-selection algorithm.	Optimizes the choice between academic background and professional experience of applicants.
DSouza (2019)		12	Explore the impact of AI, chatbots, and robots on organizational management.	Concludes that emerging technologies transform operational structures and business processes.
Birnbaum and Somers (2023)	International Journal of Organizational Analysis	9	Compare classical and modern scientific management with AI and machine learning.	Finds conceptual and methodological parallels connecting traditional scientific management with HR Analytics.
Mitravinda and Shetty (2022)		8	Design a system to assess employee turnover factors using IBM data.	Identifies critical variables predicting turnover and proposes a recommendation system for retention.
Rajpurohit et al. (2020)	International Journal of Advanced Science and Technology	8	Analyze AI use in HR Analytics to predict candidate success.	Proposes a hybrid model integrating simple tools and advanced solutions for recruitment decisions.
Peisl and Edlmann (2020)	Communications in Computer and Information Science	8	Investigate the acceptance of predictive HR Analytics among hiring managers.	Concludes that implementation improves job offer success but faces organizational resistance.
Shobhanam and Sumati (2023)	International Journal of Performability Engineering	7	Predict employee turnover using logistic regression.	Identifies key motivators such as satisfaction, work-life balance, and growth opportunities.
Shrivastava and Dhaigude (2022)	Communications of the Association for Information Systems	7	Analyze challenges in talent acquisition for ERP implementation.	Highlights candidates’ lack of commitment post-offer and proposes retention strategies.
Deepthi et al. (2024)		6	Explore the potential of Deep Learning in HR Analytics for predicting productivity.	Demonstrates that DL-based models generate accurate predictions and improve talent planning.
Cho et al. (2023)		4	Optimize human capital allocation using graph databases.	Proposes integrating competencies and projects into knowledge graphs to enhance talent management.
Ghosh and Basu (2020)	Asia Pacific Journal of Information Systems	4	Analyze challenges in talent acquisition in technology sectors.	Proposes AI-based strategies to match job descriptions with required competencies.
Kambhampati and Rao (2024)		3	Integrate machine learning and advanced analytics to reduce employee turnover.	Creates a comprehensive framework combining demographics, performance, and organizational culture to predict attrition.

Continuing the Qualitative Analysis, Table 4 synthesizes the main methodological trends identified:

**Table 4:** Main methodological trends in the 30 most cited articles on AI and data analytics in human talent management.

Approach	Frequency of use	Main applications
Machine learning	21 out of 30 studies	Prediction of turnover, performance, and satisfaction.
Explainable models (XAI)	8 out of 30 studies	Transparency and bias reduction in selection processes.
Bayesian networks	5 out of 30 studies	Optimization of complex recruitment decisions.
Prescriptive analytics	7 out of 30 studies	Automation of HR recommendations.
Big data analytics	10 out of 30 studies	Integration of massive data for People Analytics.

These results show that methodological approaches prioritize the prediction of workplace behavior and the optimization of organizational processes, with a growing emphasis on hybrid models that combine machine learning with explainable analytics.

### 3.14 General interpretation

Research on AI and data analytics in human talent management is experiencing robust growth and scientific consolidation. The results reveal a dual trend:

- First, the development of advanced predictive models that optimize strategic decision-making in human resources.
- Second, increasing attention to ethical and regulatory aspects, seeking to balance technological innovation with the protection of employee rights.

Collectively, the evidence demonstrates that AI-based talent management is shaping a new organizational paradigm, where data and advanced analytics become central elements for enhancing competitiveness, productivity, and sustainability in organizations.

## 4 Discussion

The integration between bibliometric and qualitative findings is structured through a multi-level analytical framework. Specifically, bibliometric results (Figures 2–4) identify macro-level patterns such as thematic clusters, geographic distribution, and research evolution, while the qualitative analysis (Tables 3, 4) provides micro-level insights into conceptual developments and methodological applications. This integrated approach allows the study to move beyond descriptive mapping and offer a coherent interpretation of how dominant research themes are operationalized in influential studies.

This study provides a comprehensive analysis of the evolution and current trends in the application of Artificial Intelligence (AI) and Data Analytics in human talent management between 2015 and 2025. The results reveal exponential growth in scientific production, notable methodological diversification, and the consolidation of critical thematic lines such as People Analytics, explainable predictive models, machine learning, and prescriptive analytics.

This study identifies leading countries and institutions and examines the most influential studies that have shaped the direction of this emerging field (Table 2). The evidence gathered from Figure 1 demonstrates a significant increase in publications starting in 2020, indicating that academic and business organizations have prioritized the implementation of AI tools to enhance strategic decision making in human resources. Additionally, Figure 2 shows that research is highly concentrated in India, Germany, the United States, and the United Kingdom, while Figure 3 highlights a clear thematic convergence around concepts such as machine learning, human resource analytics, and predictive analytics, reflecting a consolidated, interdisciplinary landscape.

### 4.1 Comparison with existing literature

The findings of this study align with those of other studies. Rao (Ananya and Rao, 2025) indicated that AI-driven insights improve efficiency, reduce biases, and enhance HR decision-making processes. Similarly, Rajan and Nagajothi (2024) found that AI and data analytics are transforming HR functions, including talent acquisition, employee engagement, performance evaluation, and workforce planning. Damnjanović et al. (2025) and Pryiatelchuk and Aizenberh (2024) concluded that AI also facilitates process automation, big data analysis, and personalized learning and development in global teams. However, the qualitative analysis results also present relevant differences that update the existing knowledge.

For instance, Pessach et al. (2020), one of the most cited articles, proposed a predictive analytical framework to optimize recruitment and retention processes, demonstrating that data-driven decision models significantly outperform traditional methods in terms of accuracy and efficiency. Complementarily, Margherita (2022) provides a deep conceptualization of HR analytics, structuring its applications into three key dimensions:

1. Technological enablers (digital infrastructure, automation, and Big Data).
2. Prescriptive applications (optimization of strategic decisions using AI).
3. Organizational value creation (impact on productivity, performance and sustainability).

In contrast, Giermindl et al. (2022) and Faqih et al. (2024) address the ethical risks of adopting People Analytics, noting that over-reliance on opaque algorithms can lead to issues related to data privacy, algorithmic biases, and indirect discrimination in selection processes. This emphasis on normative challenges is particularly evident in the European literature, where discussions on data governance and transparency predominate (Rajagopal, 2025). The need for XAI models (XAI) is becoming increasingly critical to ensure transparency and social responsibility (Zhai et al., 2024).

In contrast, countries such as India and the United States focus their efforts on predictive optimization and the implementation of advanced models to enhance organizational competitiveness (Sisodia et al., 2017; Deepthi et al., 2024). Furthermore, Berhil et al. (2020) reinforce the idea that the use of Big Data in HR management not only improves talent identification but also enhances organizations’ ability to adapt their strategies to changing environments.

The thematic patterns observed in Figure 3 confirm that the most influential articles prioritize the development of robust technical solutions complemented by ethical analyses and governance frameworks, indicating that the field is evolving toward a hybrid approach that combines innovation, social impact, and regulation.

### 4.2 Interpretation of main findings

The qualitative results derived from the review of the most cited articles (Table 2) identified four conceptual clusters that summarized the predominant trends in the use of AI and data analytics in human talent management.

#### 4.2.1 Cluster 1: optimization of organizational processes and people analytics

The implementation of people and HR analytics has radically transformed how organizations manage internal processes. Studies such as Margherita (2022) and Cho et al. (2023) highlight that companies integrating prescriptive analytics systems achieve improvements in workforce planning, recruitment optimization, and personalized talent retention strategies.

Likewise, Gurusinghe et al. (2021) reinforce that digitization and process automation enable organizations to respond to changing market demands. Meanwhile, Köchling et al. (2021) argue that algorithmic decision-making enhances hiring efficiency but caution that mechanisms to ensure fairness are necessary to avoid discrimination against protected groups in the hiring process.

These findings are reflected in Figure 3, where the term *HR Analytics* occupies a central node in thematic networks, underscoring its leading role in recent scientific production. Additionally, Table 2 shows that several influential studies, such as those by Pessach et al. (2020) and Giermindl et al. (2022), agree that integrating intelligent systems generates tangible improvements in organizational productivity, although it requires addressing internal resistance related to technological adoption.

#### 4.2.2 Cluster 2: implementation of predictive models and machine learning

The second trend focuses on implementing predictive models to anticipate key workplace behaviors. Sisodia et al. (2017) evaluated multiple algorithms and concluded that Random Forests and Ensemble Learning are the most effective for predicting employee turnover. In contrast, Deepthi et al. (2024) demonstrated the capability of deep neural networks to predict productivity and proposed personalized intervention strategies.

Similarly, Zhao et al. (2015) highlighted that intelligent competency normalization improves the identification of suitable candidate profiles, while Chung et al. (2023) integrated machine learning with statistical models to reduce employee attrition rates and optimize HR resource allocation.

Figure 3 supports these findings, emphasizing that *machine learning* is the most representative node in the co-occurrence network, thereby confirming its central role in scientific production. The results indicate that predictive models are transforming decision-making, enabling a shift from a reactive to a proactive approach based on robust quantitative evidence and simulations.

#### 4.2.3 Cluster 3: ethical challenges, transparency, and data governance

The expansion of AI in human resources raises complex ethical issues. Studies such as Giermindl et al. (2022) and Köchling et al. (2021) highlight that the use of opaque algorithms can amplify historical biases, affecting equal opportunity. The integration of explainable AI (XAI) models has emerged as a response to ensure transparency and fairness in decision-making processes.

Additionally, Marabelli and Lirio (2024) introduced the concept of fairness in virtual environments, proposing that the metaverse could become a key resource for improving employee experiences and promoting more inclusive and equitable practices. Meanwhile, studies such as Berhil et al. (2020) caution that AI integration must be accompanied by clear regulatory frameworks to protect privacy and establish ethical boundaries for the use of sensitive data.

#### 4.2.4 Cluster 4: technological convergence of AI, big data, and advanced analytics

The findings also indicate that modern human talent management is oriented toward adaptive intelligent systems that integrate AI, Big Data, and advanced analytics platforms. Berhil et al. (2020) and Islam et al. (2022) demonstrate that the ability to process large volumes of data enables personalized employee experiences and the design of organizational development strategies aligned with strategic objectives.

Figure 3 supports this convergence, showing that terms such as *predictive analytics*, *learning systems*, and *human resource management* exhibit strong connections, reflecting a consolidating, interdisciplinary ecosystem.

From a theoretical perspective, the results suggest that AI-driven talent management appears to be evolving toward a data-centric organizational paradigm, where predictive analytics, algorithmic decision-making, and behavioral data converge to support strategic workforce management. This transformation reflects a shift from traditional human resource management models toward algorithmically augmented decision systems, where data analytics complements managerial judgment rather than replacing it.

The convergence observed between machine learning models, People Analytics, and behavioral indicators indicates that the field is progressively moving toward an integrated socio-technical framework, combining technological capabilities with organizational theory and ethical governance mechanisms.

### 4.3 Research gaps and future opportunities

Despite significant advancements, this study identifies four critical areas requiring attention:

1. Standardization of Ethical and Regulatory Frameworks

- Studies by Giermindl et al. (2022) and Köchling et al. (2021) highlight the urgent need for clear protocols to mitigate algorithmic biases and ensure fairness.

2. Empirical Validation of Predictive Models

- Although studies like Pessach et al. (2020) and Deepthi et al. (2024) present advanced architectures, most have not been tested in real organizational settings.

3. Interdisciplinary Collaboration

- There is fragmentation between engineering, organizational psychology, and data science disciplines (Margherita, 2022), which limits the development of robust and applicable solutions.

4. Geographic Expansion of Research

- As shown in Figure 2, scientific production is concentrated in India, Germany, the United Kingdom, and the United States, while regions such as Latin America and Africa exhibit knowledge gaps, representing a strategic area for future studies.

### 4.4 Theoretical and practical implications

The findings of this research have a direct impact on three key stakeholders:

- For Organizations: Predictive models and the use of People Analytics optimize strategic talent management, improving efficiency and employee retention.
- For Academia: A solid conceptual foundation is established to drive interdisciplinary research integrating machine learning, XAI, and organizational theories.
- For Regulatory Bodies: The results underscore the need for clear public policies to ensure data protection and fairness in automated decision-making.

### 4.5 Final synthesis

The findings suggest that the application of AI and Data Analytics in human talent management is shaping a new organizational paradigm. The results reveal that although data-driven intelligent systems enable the optimization of talent selection, retention, and development, they also pose ethical, regulatory, and methodological challenges that require immediate attention.

By integrating bibliometric findings with the qualitative analysis of the 30 most cited articles, this discussion presents a comprehensive and coherent view of the field, laying the groundwork for future research and providing robust evidence for data-driven decision-making. Additionally, from a theoretical standpoint, this study contributes by proposing an integrated socio-technical framework for AI-driven talent management. Unlike previous studies that analyze technological or organizational aspects separately, this research demonstrates that effective talent management emerges from the interaction between predictive analytics capabilities, organizational decision-making processes, and ethical governance mechanisms. This integrative perspective advances the literature by conceptualizing AI not only as a technological tool but as a strategic and institutional resource embedded within organizational systems.

## 5 Conclusion

This study aimed to analyze the evolution, thematic trends, and main applications of Artificial Intelligence (AI) and Data Analytics in human talent management between 2015 and 2025 using a bibliometric approach complemented by a qualitative analysis of the 30 most cited articles in the scientific literature.

The results show exponential growth in academic production starting in 2020, reflecting the increasing interest of organizations and academics in integrating AI tools to optimize strategic decision-making in human resources (Figure 1). Additionally, it was identified that research is geographically concentrated in countries such as India, Germany, the United States, and the United Kingdom, while regions like Latin America and Africa present knowledge gaps that constitute opportunities for further study (Figure 2).

From a thematic perspective, Figure 3 and Table 2 reveal four central areas: People Analytics and process optimization, predictive models and machine learning, data governance and ethics, and technological convergence with Big Data and advanced analytics. These findings confirm that the application of AI in talent management contributes to improving employee retention, personalizing development strategies, and enhancing organizational efficiency.

Ultimately, this study provides robust evidence for data-driven decision-making and opens new lines of research in explainable artificial intelligence (XAI), intelligent automation, and the contextualization of solutions in emerging markets. Thus, AI appears to be emerging as a strategic resource for transforming human talent management, although ethical and methodological challenges persist that require priority attention.

## Data Availability

The original contributions presented in the study are included in the article/supplementary material, further inquiries can be directed to the corresponding author/s.
